# Celiac Disease as a Model of Intestinal Malnutrition: Mechanisms and Nutritional Management

**DOI:** 10.3390/nu17233741

**Published:** 2025-11-28

**Authors:** Vanessa Nadia Dargenio, Nicoletta Sgarro, Giovanni La Grasta, Martina Begucci, Stefania Paola Castellaneta, Costantino Dargenio, Leonardo Paulucci, Ruggiero Francavilla, Fernanda Cristofori

**Affiliations:** Interdisciplinary Department of Medicine, Pediatric Section, Children’s Hospital ‘Giovanni XXIII’, University of Bari “Aldo Moro”, 70126 Bari, Italy; vanessa.dargenio@unifg.it (V.N.D.); nicolettasgarro@gmail.com (N.S.); g.lagrasta94@gmail.com (G.L.G.); martinabegucci@gmail.com (M.B.); castellanetas@gmail.com (S.P.C.); costy.dargenio@gmail.com (C.D.); leonardo.paulucci92@gmail.com (L.P.); fernandacristofori@gmail.com (F.C.)

**Keywords:** celiac disease, malnutrition, deficiency, biomarkers, children

## Abstract

**Background:** In pediatric celiac disease (CD), intestinal malabsorption and the restrictive nature of a gluten-free diet (GFD) frequently result in persistent macro- and micronutrient imbalances, despite histological remission. The present review evaluates the evidence on nutritional adequacy of the GFD, identifies common deficiencies, and considers biomarker strategies and dietary recommendations to optimize growth and metabolic health. **Methods:** A narrative review of the literature was conducted, focusing on studies of nutrient intake, product composition of gluten-free foods, biomarker assessment, and clinical outcomes in children with CD. Both macronutrient (protein, fat, carbohydrate, fiber) and micronutrient (iron, vitamin D, calcium, B-vitamins, zinc, magnesium) domains were included. **Results:** Children with CD on long-term GFD demonstrate higher intake of lipids (especially saturated fat) and simple carbohydrates, alongside consistently low intake of dietary fiber and key micronutrients. Gluten-free products often exhibit lower protein content, higher glycemic index, and reduced fortification compared to gluten-containing equivalents. Nutritional deficits contribute to impaired linear growth, delayed puberty and increased metabolic risk. **Conclusions:** Nutritional adequacy of the GFD cannot be assumed in children with CD. Routine monitoring using standardized biomarker panels, combined with personalized dietary counseling and improved formulation and fortification of gluten-free products, is essential to mitigate long-term adverse outcomes. Future work should advance precision nutrition approaches and public-health initiatives to optimize dietary quality in this vulnerable population.

## 1. Introduction

An optimal diet is characterized by variety, balance, and moderation, emphasizing the intake of fruits, vegetables, whole grains, legumes, nuts, and fish, while limiting refined grains, free sugars, salt, and saturated fats [1]. Despite well-established nutritional guidelines, malnutrition persists as a widespread, multifactorial condition closely linked to numerous chronic diseases. Evidence indicates that 20–60% of hospitalized patients experience some degree of malnutrition, underscoring its clinical and public health relevance. The World Health Organization (WHO) identifies eating disorders related to both under- and overnutrition as a significant global health threat, calling for integrated strategies that combine dietary optimization, early detection, and targeted interventions to mitigate long-term consequences [2].

Malnutrition encompasses both undernutrition, manifested as wasting, stunting, and underweight, and overnutrition, including overweight and obesity [3]. Coexisting forms of malnutrition refer to the presence of more than one type of nutritional disorder in an individual, household, or population, such as the coexistence of stunting and wasting, or stunting and micronutrient deficiency [4]. The concept of the ‘double burden of malnutrition’ is a specific subcategory of coexisting forms of malnutrition, denoting the simultaneous presence of undernutrition and overnutrition, such as stunting with obesity, at the population, household, or individual level [5]. These forms are not mutually exclusive; the ‘double burden of malnutrition’ highlights the nutritional paradox increasingly observed in countries undergoing rapid nutrition transition, where both forms of malnutrition coexist and interact, often within the same person or household [6]. This duality is particularly relevant in pediatric populations, where early dietary exposures critically shape long-term health outcomes.

Among disorders most strongly associated with pediatric malnutrition is celiac disease (CD), an immune-mediated enteropathy affecting approximately 1% of the global population [6]. CD leads to selective malabsorption of macro- and micronutrients due to intestinal damage. Atrophy of intestinal villi and chronic inflammation, and consequently malabsorption, contribute to deficiencies of iron, folic acid, vitamin B12, and fat-soluble vitamins [7]. Between 20% and 67% of children with CD present signs of malnutrition at diagnosis, depending on assessment methods. Anthropometric indices such as mid-upper arm circumference (MUAC) and BMI reveal variable degrees of nutritional impairment, with stunting affecting approximately 20% of pediatric patients [8].

Clinical manifestations range from classic gastrointestinal symptoms, diarrhea, abdominal distension, and failure to thrive, to extraintestinal or subclinical presentations such as anemia, delayed puberty, and osteoporosis [9]. Iron deficiency anemia affects up to 82% of untreated pediatric CD patients, impairing cognitive and physical development, while vitamin D deficiency, often persisting despite a gluten-free diet (GFD), contributes to reduced bone mineralization and increased fracture risk [10].

Although GFD restores intestinal integrity and improves absorption, nutritional challenges frequently persist. Many gluten-free products (GFPs) are calorie-dense but poor in fiber, high-quality protein, and micronutrients [11]. Children adhering to GFDs often consume excessive amounts of ultra-processed GFPs, predisposing them to weight gain, insulin resistance, and the “double burden” of malnutrition [12].

Non-adherence to the GFD leads to increased intestinal permeability and inflammation, but even adherent children may experience deficiencies due to inadequate intake or low-grade inflammation [13].

Socioeconomic determinants further influence dietary quality [14]. Families with limited financial resources often rely on monotonous, nutrient-poor GFPs [15]. In low-resource settings, where fortified products are scarce, malnutrition prevalence is even higher. Maternal education and household income consistently predict dietary adequacy and growth outcomes in children with CD [16].

A modern approach to clinical nutrition must integrate qualitative assessment of macro- and micronutrient intake alongside total caloric evaluation [17]. This principle is especially critical in pediatrics, where optimal nutrition underpins normal growth and development.

This review examines the complex interplay between macro- and micronutrient imbalances in pediatric CD. By synthesizing current evidence, it highlights the central role of macronutrient quality in both prevention and management of malnutrition across the weight spectrum and underscores the utility of biomarkers for early detection and targeted intervention.

## 2. Macro- and Micronutrient Alterations in Pediatric CD

The GFD requires lifelong exclusion of wheat, rye, and barley [18,19]. Although it restores mucosal integrity and nutrient absorption, complete histological recovery is achieved only in a subset of patients, particularly adults [20,21]. In detail, between 5% and 26% of children with CD do not achieve histopathological remission (complete mucosal recovery) after starting a GFD, even after 1–2 years of treatment [21,22,23]. The most robust meta-analysis estimates the rate of incomplete mucosal recovery in children at approximately 35%, but rates vary widely depending on study design, duration of follow-up, and criteria for remission [24]. The main reasons for failure to achieve histopathological remission in children include inadvertent gluten exposure due to dietary non-adherence, ongoing low-level gluten contamination, more severe initial mucosal damage, and individual variability in mucosal healing rates. Some children may also have persistent immunological activity or develop partial gluten tolerance, as suggested by persistent mucosal damage despite negative serology and reported dietary adherence [23,24,25]. Serological markers (e.g., tTG-IgA) are imperfect surrogates for mucosal healing, and normalization of antibody levels does not guarantee histological remission [26].

Beyond intestinal recovery, GFD introduces distinct nutritional challenges: commercially available GFPs are often enriched in carbohydrates and fats but deficient in protein, fiber, B vitamins, iron, and folate [27,28]. These imbalances are particularly critical during growth and pubertal development, when nutritional requirements are increased. Dietary surveys reveal that many pediatric CD patients rely heavily on meat and processed GFPs while consuming fewer cereals, fruits, and vegetables [29]. Systematic reviews confirm that excessive fat and insufficient fiber and micronutrient intake are common in pediatric populations, but these deficits are exacerbated in GFD consumers [30]. Many children consume GFPs multiple times daily, increasing exposure to nutrient-poor foods [31,32].

The effects of GFD on body composition remain controversial. Some studies report beneficial outcomes, reduced body fat, improved lean mass, and catch-up growth, while others note excessive weight gain and obesity [33,34,35].

Enhanced caloric intake, combined with restored absorption after mucosal healing, may contribute to overweight and obesity [32,36]. Epidemiological studies reveal that overweight and obesity are more prevalent in pediatric CD than previously recognized, with overweight reported in 8.8–20.8% and obesity in 0–6% at diagnosis, increasing after GFD initiation to 9.4–21% and 0–8.8%, respectively [37]. Findings remain inconsistent, as some analyses indicate no significant increase in obesity risk with GFD adherence [38,39].

In summary, children with CD exhibit both macro- and micronutrient imbalances, stemming from pre-diagnosis malabsorption and long-term dietary inadequacies. The coexistence of insufficient fiber and micronutrient intake with excessive lipid and carbohydrate consumption highlights a dual risk of undernutrition and overweight, underscoring the need for continuous nutritional surveillance and individualized dietary counseling.

### 2.1. Specific Macronutrient Deficiencies in Pediatric CD

Macronutrients, proteins, carbohydrates, and fats play a pivotal role in health and disease, not only through their quantitative contribution but also through their qualitative composition and distribution within the diet [40]. Alterations in macronutrient balance can exert profound effects on key physiological systems, influencing hormonal regulation, immune responses, basal metabolic rate, and systemic inflammation. The following sections will explore these components in greater detail.

### 2.2. Protein Intake

Protein malnutrition in CD results from crypt hyperplasia, which reduces enzymatic activity and absorptive surface area, limiting amino acid assimilation [7]. Consequently, untreated CD patients often exhibit decreased serum albumin and other protein markers, indicating compromised protein status [41]. Although the GFD promotes mucosal recovery, it may exacerbate protein insufficiency in longitudinal follow-up, as GFPs generally contain low-quality proteins and high saturated fat levels [42]. This imbalance contributes to suboptimal protein intake and metabolic dysregulation. Diets dominated by processed meats may intensify inflammation, whereas those emphasizing legumes, fish, and plant-based proteins improve amino acid balance and reduce inflammatory stress [43]. Findings on protein intake in CD remain inconsistent. Mariani et al. reported elevated intake among adolescents [44], while Shepherd and Gibson [45] and Van Hees et al. [29] observed reduced vegetable protein consumption. These discrepancies likely reflect regional differences in diet and GFP composition. Recent data by Ekşi et al. confirmed lower absolute and relative protein intake in pediatric CD patients compared with controls, frequently below dietary reference values [34].

Overall, evidence indicates that although protein inadequacy is not universal, a significant proportion of pediatric patients remain at risk of insufficient intake.

### 2.3. Carbohydrate Intake

Carbohydrate intake in CD is often characterized by refined gluten-free starches, such as rice and corn flour, with high glycemic indices (GIs), leading to rapid digestion and postprandial glucose surges that may promote insulin resistance and metabolic syndrome. Insufficient soluble fiber further reduces gastrointestinal motility and alters gut microbiota composition, compromising intestinal barrier function and inducing chronic immune activation [33].

Current dietary strategies recommend incorporating naturally gluten-free whole grains and pseudocereals, which have lower GIs and higher fiber content. These foods improve glycemic control, enhance satiety, and support microbial diversity [43]. Nevertheless, population studies consistently report excessive sugar intake and inadequate fiber consumption among children with CD compared with healthy peers [29].

Gluten exclusion may also alter carbohydrate metabolism. Experimental evidence suggests that gluten inhibits starch hydrolysis; thus, its removal may increase glycemic responses to carbohydrate ingestion [46]. Indeed, many GFPs have higher GIs than gluten-containing (GC) equivalents [46], and pediatric studies confirm elevated glycemic values in CD compared with controls [47]. This finding is particularly relevant for patients with concurrent type 1 diabetes, a common comorbidity in CD [48].

Although data remain limited, available evidence consistently indicates heightened glycemic exposure in pediatric CD [49]. Further longitudinal studies are required to clarify long-term metabolic risks and guide evidence-based dietary recommendations.

### 2.4. Fat Intake

In untreated CD, lipid metabolism is disrupted due to fat malabsorption, resulting in deficiencies of essential omega-3 and omega-6 polyunsaturated fatty acids (PUFAs), crucial for membrane integrity and inflammation control [46]. Conversely, adherence to a GFD often leads to excessive saturated fat intake and insufficient unsaturated fat consumption, producing an atherogenic lipid profile with elevated LDL and reduced HDL cholesterol [50].

Comparative analyses of GFPs and GC foods show marked nutritional disparities. For example, Spanish data revealed that gluten-free breads contain nearly twice the total fat and three times the saturated fat of standard breads, with similar trends in pasta and bakery items [51]. These modifications, designed to enhance texture and palatability, have significant nutritional implications. While some studies report higher fat intake in children with CD than in controls [52], others found no difference [46,53]. Notably, excessive fat intake surpassing dietary recommendations has been documented in both CD and non-CD pediatric populations [30,54].

Beyond caloric excess, high-fat consumption is associated with a greater prevalence of overweight and obesity among adolescents with CD on GFDs [55] and an elevated risk of metabolic syndrome and other systemic diseases [56,57]. Although causality remains uncertain, the combination of high-fat GFPs, restored nutrient absorption, and modern processed-food habits likely contributes to this trend. Nutritional interventions emphasizing unsaturated fat sources, such as extra virgin olive oil, nuts, and oily fish, are therefore essential to re-establish lipid balance and reduce long-term cardiometabolic risk [58].

### 2.5. Fiber Intake

The fiber content of GFPs is generally low due to reliance on refined flours and starches, in which the outer grain layers, rich in fiber, are removed during processing [59]. Studies in adults show that GFDs provide less dietary fiber than GC diets [60]. In children, however, fiber intake typically falls below recommendations regardless of CD status, suggesting a broader dietary shortfall rather than a condition-specific deficit [60]. Mariani et al. reported significantly lower fiber intake among adolescents with CD compared to healthy peers [44], though most evidence points instead to a Westernized dietary pattern, marked by low intake of fiber-rich plant foods and whole grains in favor of refined, processed products. Consequently, suboptimal fiber intake in pediatric CD likely reflects both the limited nutritional value of GFPs and general population-level trends.

Low fiber intake has important clinical consequences, including impaired satiety, slower gastrointestinal transit, and reduced microbial diversity [46]. Recent GFP innovations incorporating pseudocereals have improved fiber content to levels approaching those of traditional wheat-based products [61], though adoption among pediatric patients remains limited.

Cross-sectional data from Babio et al. showed that both CD and non-CD children consume inadequate fiber and few fruits and vegetables [62]. Seasonal variations further complicate interpretation. While two studies reported lower fiber intake in GFD consumers [63,64], the majority confirm that both groups consistently fail to meet dietary fiber recommendations.

Collectively, these findings indicate that inadequate fiber intake is not exclusive to pediatric CD but is exacerbated by dependence on refined GFPs.

Table 1 summarizes macronutrient alterations in pediatric CD, highlighting the dual impact of intestinal malabsorption and suboptimal composition of GFPs on overall nutritional balance and metabolic health.

## 3. Specific Micronutrient Deficiencies in Pediatric CD

Micronutrients are essential to physiological homeostasis, yet many must be acquired through diet as they cannot be synthesized endogenously. Many studies evaluating nutrient intake in CD rely on nutritional data derived from commercial GFP labels, yet the inconsistent reporting of micronutrient composition introduces the potential for underestimation of actual intake in the literature.

### 3.1. Iron Deficiency

Iron deficiency is one of the most common extraintestinal manifestations of CD, presenting either as overt iron deficiency anemia (IDA) or as subclinical depletion. Ferritin is the most reliable biomarker of iron status, though its interpretation may be confounded by inflammation. Iron deficiency, with or without anemia, is frequently observed in pediatric CD, particularly at diagnosis. One study reported that 73.3% of affected children showed iron deficiency, with the highest prevalence among infants and children over 12 years of age [65]. Similarly, Habib et al. found anemia in 30.1% of children with CD, of whom 21.6% had confirmed IDA, while a regional study from Southern Punjab, Pakistan, revealed that 17.4% of children with IDA were subsequently diagnosed with CD [66].

Iron deficiency may be overt or subclinical and can, in some cases, represent the sole manifestation of CD, especially in atypical or silent forms lacking gastrointestinal symptoms [67]. Serological testing for CD-specific antibodies, followed by duodenal biopsy when indicated, remains the diagnostic gold standard [68]. In one cohort of children with moderate-to-severe IDA, 10.5% tested positive for CD serology, and 3.9% were confirmed histologically [69].

Most pediatric patients normalize ferritin levels within 12 months of GFD initiation, even without iron supplementation. Persistent deficiency despite compliance may require pharmacologic iron replacement. Regular monitoring of hemoglobin, ferritin, and serum iron levels is essential for effective management and to prevent recurrence [70]. In summary, hemoglobin, ferritin, and serum iron should be monitored every 1–3 months during active treatment for IDA, and every 3–6 months for iron deficiency without anemia. After correction, regular monitoring should be continued to prevent recurrence, adjusting frequency based on underlying risk factors and disease activity [70].

### 3.2. Vitamin D Deficiency

Vitamin D deficiency is markedly more prevalent in children with CD than in healthy controls. Meta-analyses report that serum 25-hydroxyvitamin D_3_ levels are, on average, 5.77 nmol/L lower in children with CD [71]. In a Kuwaiti cohort, 31% of affected children exhibited growth stunting and 20.8% had a low BMI-for-age, with vitamin D deficiency identified as a key factor [72]. Similarly, a cross-sectional study found that 31.5% of children positive for anti-tissue transglutaminase antibodies were vitamin D deficient, highlighting the need for systematic screening [73].

The primary mechanism is intestinal malabsorption, which reduces uptake of fat-soluble vitamins, including vitamin D [74]. Other contributors include insufficient dietary intake and limited sunlight exposure, both of which are frequently observed in pediatric populations with CD [75]. Socioeconomic factors, such as lower household income and maternal education, further increase the risk of vitamin D deficiency and growth impairment [72]. Routine measurement of serum 25(OH)D levels is therefore recommended at diagnosis and during follow-up to ensure sufficiency and to optimize bone health [19].

Several studies have shown rising 25(OH)D_3_ levels after GFD initiation [75,76]. Moreover, fortification of GFPs with vitamin D has demonstrated additional benefits for serum vitamin D concentrations and bone health in children with CD [77].

### 3.3. Zinc

Zinc deficiency is highly prevalent among children with CD, including those adhering to a GFD. In one study, 91% of pediatric CD patients on a GFD exhibited low serum zinc levels, with median concentrations significantly lower than those of healthy controls. The pathophysiology involves intestinal epithelial damage that impairs zinc absorption and increases fecal zinc losses [8]. Untreated CD is consistently associated with reduced serum zinc concentrations compared with non-CD controls, indicating that the enteropathy itself drives systemic depletion [78]. Zinc plays an essential role in epithelial integrity, immune competence, and growth. Deficiency can cause stunted growth, immune dysfunction, and increased susceptibility to infections such as diarrhea and pneumonia [79,80,81,82].

### 3.4. Magnesium

Children with CD, particularly those with concurrent malnutrition, are at elevated risk of magnesium deficiency due to both insufficient dietary intake and impaired intestinal absorption. This deficiency contributes to poor growth and overall health impairment [72]. Magnesium is crucial for bone mineralization, and its deficiency has been associated with osteoporosis and reduced bone density [83]. Severe cases may present with neuromuscular symptoms such as spasms, tremors, and convulsions, which resolve following repletion [83]. In addition to musculoskeletal and neurological effects, magnesium deficiency promotes systemic inflammation and oxidative stress, potentially worsening intestinal injury and metabolic disturbances in CD [84]. Ensuring adequate magnesium intake through diet or supplementation is therefore essential. Regular monitoring of serum magnesium levels is recommended, especially in children with persistent malabsorption or inadequate intake [85].

### 3.5. B Vitamins

Among the most clinically relevant micronutrient deficiencies in untreated CD are those of vitamin B_12_ and folate, both essential for hematopoiesis, neurological function, and cellular growth. Although the terminal ileum, the primary site of vitamin B_12_ absorption, is typically less affected in CD, deficiency has been reported in up to 41% of patients [86]. The underlying mechanisms include the CD-driven mucosal injury and impaired pancreatic exocrine function, both of which compromise cobalamin absorption [87,88].

In children, vitamin B_12_ deficiency can cause developmental delay, cognitive impairment, and megaloblastic anemia [89,90].

Notably, up to 16.3% of children with severe acute malnutrition show vitamin B_12_ deficiency, aggravating clinical outcomes [91].

Folate deficiency is equally concerning in pediatric CD and primarily results from the restrictive nature of GFDs. Many GFPs lack mandatory folate fortification, as observed in countries such as Canada, where folate intake among GFD consumers is significantly lower than in those consuming fortified wheat-based foods [92]. Folate deficiency impairs DNA synthesis and cell division and elevates homocysteine levels, a known risk factor for cardiovascular and skeletal disorders [93]. Autoimmune enteropathy in CD further reduces folate absorption [94]. Serum and erythrocyte folate assessments are reliable for diagnosing deficiency in children with CD [95]. While adherence to a GFD generally improves absorption, persistent deficiencies require supplementation. A strict GFD remains the cornerstone of management, often leading to gradual normalization of vitamin B_12_ and folate levels [86,96]. Nevertheless, continued monitoring is essential, as deficiencies may persist despite mucosal recovery, and supplementation may be necessary to prevent anemia, growth failure, and neurocognitive impairment [97].

Table 2 summarizes the principal micronutrient deficiencies identified in pediatric CD, outlining their pathophysiology, prevalence, clinical implications, and evidence-based management strategies.

## 4. Consequences of Macro- and Micronutrient Malnutrition in Pediatric CD

Children with CD are especially vulnerable to the adverse effects of macronutrient malnutrition. Chronic deficiencies can impair linear growth and delay puberty, with lasting effects on physical and psychosocial development [98]. During this critical developmental period, inadequate intake of energy, protein, and essential vitamins and minerals can also compromise bone health, immune function, and neurocognitive outcomes.

The growing consumption of ultra-processed GFPs, typically high in sugar and saturated fat but low in fiber and micronutrients, further increases metabolic risks and contributes to gut microbiota dysbiosis [43]. Several studies report rises in BMI and fat mass among pediatric CD patients after starting a GFD [99]. Więch et al. observed greater increases in weight, BMI, and fat mass in adherent children [100], while Kabbani et al. found that 15.8% of adults shifted from normal or low BMI to overweight following GFD initiation [101]. Conversely, Ukkola et al. noted normalization of weight in previously underweight patients, highlighting heterogeneity in outcomes [102].

Therefore, dietary counseling should focus on limiting processed GFPs and emphasizing naturally gluten-free whole foods.

Effective management of macronutrient malnutrition in CD requires a multidisciplinary approach involving dietitians, gastroenterologists, and primary care providers. Beyond gluten exclusion, nutritional education should promote: (a) naturally gluten-free whole grains (e.g., quinoa, millet, buckwheat) to enhance fiber and complex carbohydrate intake; (b) diverse, high-quality protein sources such as legumes, fish, and plant-based proteins to optimize amino acid balance and reduce inflammation; (c) replacement of saturated fats with monounsaturated and polyunsaturated fats from olive oil, nuts, seeds, and fatty fish; (d) reduced consumption of processed GFPs high in sugar and unhealthy fats. Personalized interventions should be guided by anthropometric, biochemical, and dietary assessments. When dietary adjustments are insufficient, targeted supplementation with key nutrients may be indicated.

## 5. Nutritional Profile of GC and GFPs

Traditional GC staples such as wheat, barley, and rye breads provide essential macro- and micronutrients, including carbohydrates (42.7–51.9 g/100 g), protein (8.5–12.5 g/100 g), calcium, iron, zinc, magnesium, phosphorus, potassium, and B vitamins [103,104,105]. In contrast, rice- and corn-based GFPs are typically deficient in protein, fiber, and folate [29]. To replicate gluten’s structural properties, manufacturers often add starches, fats, dairy or egg proteins, and hydrocolloids [29], thereby increasing caloric density and GI [29,106], which may lead to adverse metabolic outcomes [107,108]. Moreover, most GFPs lack micronutrient fortification, resulting in reduced vitamin and mineral intake.

International surveys across multiple continents reveal substantial variability in GFP nutrient composition [109,110,111,112,113,114,115,116]. Energy content is inconsistent; some studies report equivalence with GC products [29], while others find lower or higher values depending on category [109,115]. Fat content is frequently elevated [29,113], though findings vary [112,117]. Saturated fat levels are similarly inconsistent [29,118]. Carbohydrate and sugar data are heterogeneous, with several studies reporting higher sugar content in GFP breads and flours [111,113].

Fiber and protein deficits remain the most consistent findings. Multiple analyses document lower fiber in GFPs [110], while others note category-specific variation [111,115]. Protein content is uniformly reduced, with GFP bread containing up to 30% less protein than wheat bread [112,113,115,116]. Alternative ingredients such as quinoa, amaranth, buckwheat, and legumes can improve protein and micronutrient profiles, but their use remains limited. Reformulation with unsaturated fats and vitamin–mineral fortification is needed to align with WHO/FAO guidelines limiting saturated fats to <10% of total energy.

Despite recent improvements, most GFPs still contain more fat and carbohydrate, higher glycemic potential, and less protein and micronutrients than GC counterparts [119]. These imbalances, coupled with inadequate fortification, compromise dietary adequacy in CD patients, especially children and those with restrictive diets [120]. Bread and pasta exemplify these disparities: GFP bread is higher in fat but lower in protein [116], while GFP pasta, often made from rice or corn starch, has an elevated GI [120]. Consequently, GFPs cannot be considered nutritionally equivalent to GC products [121].

Reformulation efforts should focus on protein enrichment, improved lipid profiles, and micronutrient fortification, with increased use of pseudocereals and legumes. Until such strategies are widely adopted, clinicians should encourage patients to prioritize naturally gluten-free, nutrient-dense foods and to limit processed GFP consumption.

## 6. Gut Microbiota and GFD in CD

Diet plays a central role in modulating gut microbiota composition and function, thereby influencing host physiology through multiple mechanisms [122]. Microbiota development progresses from birth to adulthood, stabilizing in mature individuals, although dietary changes, gastrointestinal disease, and antibiotics can significantly alter microbial balance [122]. Two main mechanisms underlie dietary modulation: competition among microbial species for substrates and diet-dependent variations in pH, bile salts, and micronutrient availability [122].

Individuals with CD exhibit distinct microbial profiles compared with healthy controls, with dysbiosis often persisting despite adherence to a GFD [123]. Reductions in beneficial taxa such as *Bifidobacterium longum* and *Lactobacillus* spp. are accompanied by increases in *Enterobacteriaceae* spp. [123]. These alterations reflect reduced polysaccharide intake during GFD adherence [124]. Since undigested polysaccharides normally reach the distal colon as fermentation substrates, their absence promotes competition and overgrowth of opportunistic species [124]. De Palma et al. documented significant declines in *Bifidobacterium* spp., *Clostridium lituseburense* group, *Fecalibacterium prausnitzii*, *Lactobacillus* spp., and *Bifidobacterium longum*, together with increases in *Escherichia coli*, Enterobacteriaceae, and *Bifidobacterium angulatum* after one month of GFD exposure, suggesting that the GFD may inadvertently promote dysbiosis rather than restore microbial balance. Dysbiotic patterns compromise mucosal defense and foster chronic inflammation [124]. Polysaccharide fermentation generates short-chain fatty acids (SCFAs) that inhibit enterobacteria; low fiber intake diminishes SCFA production, reducing this protective effect. Conversely, fiber enrichment supports SCFA synthesis and microbial diversity [124].

The gut microbiota influences nutrient metabolism, xenobiotic degradation, mucosal barrier integrity, pathogen defense, and immune regulation [125]. Microbial composition is shaped not only by diet but also by birth delivery mode and antibiotic exposure [126]. Notably, diet-induced microbial shifts occur rapidly: David et al. demonstrated that dietary modification alters microbiota within days [127]. Persistent dysbiosis despite gluten withdrawal remains a major concern. Golfetto et al. found that CD patients exhibit microbial imbalance even under a GFD [128]. Whether dysbiosis precedes CD onset or results from gluten exclusion remains uncertain [128,129]. De Palma et al. similarly observed microbial depletion after one month of GFD in healthy adults, emphasizing the microbiota-disruptive potential of gluten exclusion [124].

Adjunctive interventions are therefore needed to mitigate GFD-related dysbiosis. Probiotic supplementation may restore beneficial taxa and enhance microbial resilience [130]. Combining probiotics with fiber- and polysaccharide-rich naturally occurring GFPs could promote microbial diversity and functionality, thereby improving clinical outcomes in CD patients [131]. We compare the macronutrient and micronutrient composition of GFPs and GC products, highlighting the consistently lower protein and micronutrient density, higher fat content, and elevated GI commonly observed in gluten-free formulations (Table 3).

## 7. Addressing Malnutrition in CD: Integrated Strategies and Future Perspectives

Malnutrition remains a critical clinical and public health issue in CD, driven by both intestinal malabsorption and the restrictive nature of the GFD. Effective management demands a multifactorial approach integrating clinical monitoring, supplementation, and policy initiatives to sustain metabolic health and support optimal growth. While traditional anthropometry and dietary recalls provide valuable insights, they are insufficient to capture the biochemical complexity of malnutrition. The incorporation of molecular and biochemical biomarkers, encompassing gut microbiota composition, systemic inflammatory mediators (e.g., IL-6, CRP), and metabolic indices, offers earlier and more precise detection of deficiencies, enabling tailored interventions. Longitudinal studies remain essential to elucidate the long-term effects of GFD adherence on bone density, body composition, and micronutrient balance in both pediatric and adult cohorts.

Dietary optimization continues to be the cornerstone of therapy. A nutritionally adequate GFD supported by fortified GFPs and appropriate supplementation can mitigate common deficiencies. Prioritizing fortification with iron, folate, vitamin D, and calcium is particularly important to prevent complications such as anemia, osteopenia, and delayed growth. Emerging advances in precision medicine are redefining nutritional care in CD. Integration of genetic, microbiome, and metabolomic profiling enables the identification of individuals predisposed to nutrient malabsorption or altered metabolism, facilitating personalized supplementation strategies.

Combating malnutrition in CD requires coordinated action among clinicians, researchers, policymakers, and the food industry. Policymakers and the food industry must prioritize mandatory fortification of GFPs and reformulate products to improve protein quality, fiber, and lipid profiles, ensuring nutritional equivalence with GC foods.

Ultimately, integrating clinical, molecular, and nutritional data into comprehensive care frameworks represents the future of CD management, shifting the focus from symptom control to preventing long-term metabolic and nutritional sequelae.

## 8. Limitations and Future Directions

Despite growing interest in the nutritional consequences of pediatric CD, current evidence is limited by methodological inconsistencies, product variability, and a lack of long-term data.

Macronutrient intake data remain inconsistent, particularly for protein and fat, due to regional dietary differences, evolving GFP formulations, and non-uniform assessment tools. While fiber intake is often lower in CD populations, this reflects a broader dietary inadequacy common to children overall, exacerbated in CD by reliance on refined GFPs.

Commercial GFPs frequently exhibit suboptimal nutritional profiles, high in saturated fats and simple sugars, yet low in protein, fiber, and key micronutrients (iron, folate, B vitamins). Fortification is uncommon, and labeling is often incomplete, limiting accurate intake assessment. The integration of nutrient-dense ingredients like legumes and pseudocereals is hindered by cost and processing constraints.

Longitudinal studies are critically lacking. The long-term impact of GFD on growth, bone health, body composition, and metabolic risk remains underexplored, particularly in high-risk subgroups such as children with concurrent type 1 diabetes. Persistent dysbiosis despite GFD adherence further raises questions about microbiota-related consequences.

Finally, multi-omics and precision nutrition approaches remain underutilized but represent key future directions for optimizing individual care and public health strategies.

## 9. Conclusions

Macronutrient and micronutrient imbalances remain critical challenges in the nutritional management of pediatric CD. Ensuring nutritional adequacy in pediatric CD extends beyond gluten exclusion. GFPs are frequently suboptimal, characterized by low protein quality, excessive saturated fat, insufficient dietary fiber, and limited fortification with essential vitamins and minerals. These deficiencies, particularly involving iron, vitamin D, calcium, and B vitamins, contribute substantially to growth impairment, delayed pubertal development, and increased long-term metabolic and skeletal risk.

Precision-based nutritional strategies, integrating targeted supplementation, dietary diversification, and, when necessary, specialized nutritional support, should form the foundation of individualized management. Systematic use of biochemical and molecular monitoring enables early detection of deficiencies.

Collaborative efforts among clinicians, dietitians, researchers, and the food industry should focus on reformulating GFPs with improved fortification and composition to promote balanced nutrition and lifelong metabolic and developmental health in children with CD.

## Figures and Tables

**Table 1:** Specific macronutrient deficiencies and imbalances in pediatric CD: pathophysiology, prevalence, and clinical implications.

Macronutrient	Mechanism of Alteration	Prevalence/Key Findings	Clinical Consequences	Dietary and Clinical Management
Protein	Intestinal damage; GFPs low in high-quality protein; imbalance from ↑ fat and sugar intake.	↓ Protein intake in many CD cohorts; ↓ vegetable protein in long-term GFD users [29]; some heterogeneity across studies.	Hypoproteinemia; ↓ muscle mass; impaired growth; delayed healing.	Emphasize mixed protein sources (legumes, fish, lean meat); improve amino acid profile via pseudocereals; consider protein-fortified GFPs.
Carbohydrates	Reliance on refined starches (rice, corn) with high glycemic index; loss of gluten-mediated inhibition of starch hydrolysis; ↓ intake of carbs.	↑ glycemic index in CD children vs. controls [47]; ↑ sugar intake and ↓ fiber across [29].	Postprandial hyperglycemia, insulin resistance; metabolic syndrome risk; altered gut microbiota.	Promote naturally gluten-free whole grains (quinoa, amaranth, buckwheat); limit refined GFPs; integrate prebiotic fibers.
Fats	Intestinal damage; high-fat GFPs to improve palatability; ↑ saturated fat intake on GFD.	Fat intake above recommendations in most pediatric CD cohorts [52,55]; GFPs contain ~2× more total fat than GC analogs.	Dyslipidemia (↑ LDL, ↓ HDL), obesity, metabolic syndrome; low PUFA status linked to inflammation.	Prioritize unsaturated fats (olive oil, nuts, fish); limit processed GFPs; regular lipid profile monitoring.
Fiber	Use of refined flours and starches ↓ fiber density; removal of outer grain layers during milling; limited intake of plant-based foods.	Fiber intake consistently below dietary reference values; ↓ in CD adolescents vs. controls [46].	Constipation; Dysbiosis; ↑ metabolic and cardiovascular risk.	Encourage legumes, fruits, vegetables, and pseudocereals; reformulate GFPs with higher-fiber grains.

CD—Celiac disease; GFD—Gluten-free diet; GFP—Gluten-free product; GC—gluten containing; PUFA—Polyunsaturated fatty acids; LDL—Low-density lipoprotein; HDL—High-density lipoprotein; ↓, reduce; ↑, increase

**Table 2:** Specific micronutrient deficiencies in pediatric CD: prevalence, mechanisms, and clinical implications.

Micronutrient	Mechanism of Deficiency	Prevalence/Key Findings	Clinical Consequences	Management and Monitoring
Iron	Intestinal damage; chronic mucosal inflammation; ↓ intake from unfortified GFPs.	Iron deficiency or anemia	Iron deficiency, anemia, fatigue; impaired growth and cognition.	GFD restores mucosal absorption; ferritin and Hb should be monitored; oral/IV supplementation if persistent.
Vitamin D	Intestinal damage; limited intake and sunlight exposure.	31–32% deficient in pediatric cohorts; linked to low BMI and stunting.	Rickets, osteopenia, delayed growth, increased fracture risk.	Routine 25(OH)D testing; supplementation and fortified GFDs recommended.
Zinc	Intestinal damage; fecal loss; chronic inflammation.	Deficiency in up to 91% of CD children on GFD.	Growth retardation; impaired immunity, recurrent infections.	zinc supplementation, and serum level monitoring.
Magnesium	Intestinal damage; ↓ dietary intake; fecal loss; oxidative stress increases demand.	Frequent in malnourished CD children; associated with bone and neurological complications.	Muscle spasms, tremors, convulsions; reduced bone density; inflammation.	Dietary enrichment (legumes, nuts, seeds) or supplements; serum magnesium monitoring.
Vitamin B_12_	Intestinal damage; pancreatic insufficiency; ↓ intake in GFD.	Deficiency in up to 41% of pediatric CD cases; higher prevalence in severe malnutrition.	Megaloblastic anemia; neurocognitive impairment; developmental delay.	Vitamin B_12_ supplementation and serum level monitoring.
Folate (Vitamin B_9_)	Intestinal damage; ↓ intake from non-fortified GFPs.	↓ dietary folate in GFD consumers; associated with ↑ homocysteine and anemia.	Megaloblastic anemia; growth failure; cardiovascular and skeletal risk.	Encourage folate-rich foods (legumes, leafy greens) or supplementation; monitor serum folate and homocysteine.

CD—Celiac disease; GFD—Gluten-free diet; GFP—Gluten-free product; Hb—Hemoglobin; 25(OH)D—25-hydroxyvitamin D; ↓, reduce; ↑, increase.

**Table 3:** Comparative nutritional characteristics of GFPs and GC products.

Nutrient/Property	GC Products	GFPs
Energy (kcal/100 g)	230–280	Similar or slightly lower; varies by product type
Protein (g/100 g)	8.5–12.5 (wheat bread)	↓ 20–40%; average 5–8 g/100 g
Total Fat (g/100 g)	2.0–4.5	↑ 50–100% higher; often 5–9 g/100 g
Saturated Fat (g/100 g)	0.5–1.5	↑ or comparable depending on formulation; often >2 g/100 g
Carbohydrates (g/100 g)	42–52	Similar or ↑ (especially in starch-based GFPs)
Sugars (g/100 g)	1.0–3.5	Variable; ↑ in breads, cakes, and mixes
Dietary Fiber (g/100 g)	3–7 (wholegrain); 1–2 (white bread)	↓ in most GFPs; occasionally ↑ in fiber-enriched products
Glycemic Index (GI)	50–70 (moderate)	↑ High; often >80 for rice/corn-based GFPs
Micronutrient Content	Fortified with iron, folate, B vitamins, minerals	↓ Unfortified; lower levels of iron, calcium, zinc, folate
Fortification Status	Mandatory in most countries	Rarely fortified; labeling inconsistent
Pseudocereal/Legume Inclusion	Rare in standard formulations	Emerging use (quinoa, amaranth, buckwheat, legumes) improves protein and fiber content
Overall Nutritional Quality	Balanced macronutrient distribution; fortified	Often imbalanced: ↑ fat, ↑ GI, ↓ protein, ↓ micronutrients

GFPs—Gluten-Free Products; GC—Gluten-Containing Products; GI—Glycemic Index; ↓, reduce; ↑, increase.

## Data Availability

No new data were created or analyzed in this study. Data sharing is not applicable to this article.

## Abbreviations

The following abbreviations are used in this manuscript:

BMI	Body Mass Index
CD	Celiac Disease
CRP	C-Reactive Protein
ESPGHAN	European Society for Paediatric Gastroenterology
FAO	Food and Agriculture Organization
GC	Gluten-Containing
GFD	Gluten-Free Diet
GFP	Gluten-Free Product
GI	Glycemic Index
Hb	Hemoglobin
HDL	High-Density Lipoprotein
HSA	Human Serum Albumin
ID	Iron Deficiency
IDA	Iron Deficiency Anemia
IL-6	Interleukin-6
LDL	Low-Density Lipoprotein
MS	Metabolic Syndrome
MUAC	Mid-Upper Arm Circumference
PUFA	Polyunsaturated Fatty Acids
SCFA	Short-Chain Fatty Acids
WHO	World Health Organization
